# Implications of an Incidental Pulmonary Arteriovenous Malformation

**DOI:** 10.1177/2324709616637190

**Published:** 2016-03-08

**Authors:** Van K. Holden, Nirav G. Shah, Avelino C. Verceles

**Affiliations:** 1 University of Maryland Medical Center, Baltimore, MD, USA

**Keywords:** arteriovenous malformations, hereditary hemorrhagic telangiectasia

## Abstract

*Introduction.* Pulmonary arteriovenous malformations (PAVMs) have been associated with life-threatening complications, such as stroke and massive hemoptysis, thus posing significant morbidity if left untreated. We report a case of an incidental finding of a PAVM in a trauma patient newly recognized to have suspected hereditary hemorrhagic telangiectasia (HHT). *Case Description.* A 34-year-old man with a history of recurrent epistaxis presented with a sudden fall associated with seizure-like activity. Trauma imaging showed a large subdural hematoma and, incidentally, a serpiginous focus within the right upper lobe with a prominent feeding artery consistent with a PAVM. The patient was diagnosed with a simple PAVM related to possible or suspected HHT, an autosomal dominant trait with age-related penetrance. He underwent a pulmonary arteriography of the right upper and lower lobe with the use of a microcatheter system; however, the PAVM could not be visualized. Thus, he was managed medically. The patient was educated on the need for prophylactic antibiotics prior to dental procedures and surveillance imaging. *Discussion.* Our case highlights the importance of obtaining a complete past medical and family history in young patients with a history of recurrent epistaxis to elicit features of HHT. The diagnosis can be made clinically and directly affects family members, who would otherwise not receive appropriate screening.

## Introduction

PAVMs are caused by abnormal communications between pulmonary arteries and veins. They can be single or multiple, unilateral or bilateral, and simple or complex. Risks of untreated PAVMs include stroke, cerebral abscess, migraine, pulmonary hypertension, and hemorrhage.^1,2^ Most PAVMs are associated with HHT, an autosomal dominant trait that affects the development of the vasculature. Unfortunately, HHT is underdiagnosed, leading to entire families being unaware of the available screening and treatment.^3-5^ We report the case of a young man with a history of epistaxis who presented with a traumatic fall and was found to have an incidental PAVM related to suspected HHT. The case highlights the importance of obtaining a complete medical and family history to elicit features concerning for this hereditary condition.

## Case Presentation

A 34-year-old man with a history of recurrent epistaxis presented with a sudden fall associated with seizure-like activity. He was brought to our trauma center and was found to have a large subdural hematoma. The patient did not have any medical history except for intermittent nosebleeds since childhood. He smoked a pack of cigarettes daily, drank alcoholic beverages socially, and used marijuana. Family history revealed that his twin brother also suffered from nosebleeds, and the patient’s mother had a transient ischemic attack of unknown etiology at the age of 32.

On admission, vital signs were significant for a heart rate of 47 beats per minute and a respiratory rate of 40 breaths per minute with a Glasgow Coma Scale (GCS) score of 6. The patient was immediately intubated. He had a fixed, dilated 7-mm right pupil on examination. Preliminary blood work showed an elevated total leukocyte count (12 × 10^9^/L) with an otherwise normal complete blood count and coagulation profile. A blood ethanol level was markedly elevated at 56.88 mmol/L. A computed tomography (CT) scan of the head revealed a large, right subdural hematoma with 1.6-mm leftward midline shift, uncal herniation, and obstructive hydrocephalus. Emergent craniotomy with evacuation of the subdural hematoma was performed. Subsequently, a brain CT venogram demonstrated 3 sites of nonocclusive thrombus within the superior sagittal sinus and the right transverse sinus. There were no cerebral arteriovenous malformations identified on imaging.

**Figure 1.**
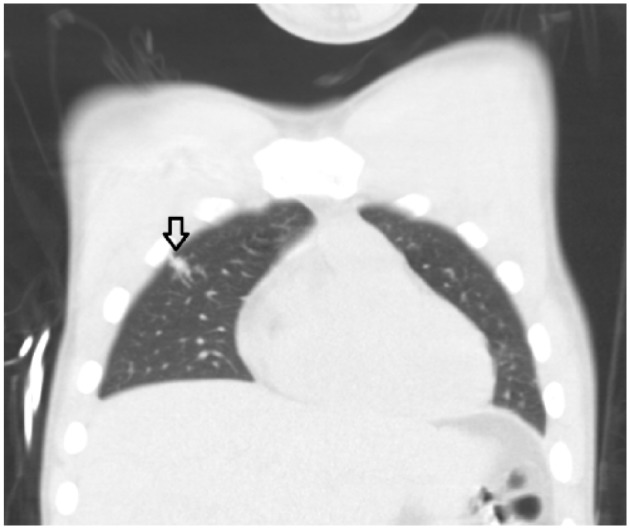
Coronal section of CT chest shows a serpiginous focus within the right upper lobe.

**Figure 2.**
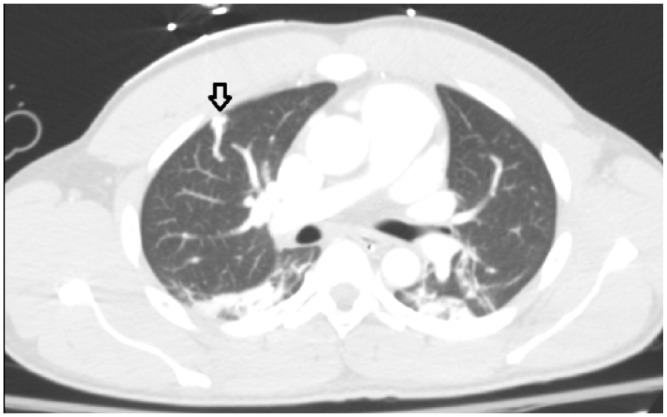
CT chest with contrast shows a focus within the inferior right upper lobe with a prominent feeding artery 2.9 mm in diameter, consistent with an arteriovenous malformation.

Incidentally, trauma imaging showed a serpiginous focus within the right upper lobe with a prominent feeding artery and vein arising from the pulmonary artery and pulmonary veins, respectively, consistent with a pulmonary arteriovenous malformation (PAVM; Figure 1). The feeding artery measured 2.9 mm in diameter (Figure 2). The patient did not have any hemoptysis or cyanosis. The trauma imaging protocol at our institution consists of a CT head without contrast and a CT chest, abdomen, and pelvis with contrast using a 64-slice multidetector CT scan. A case-matched series has found that patients who underwent immediate total-body CT scanning had a significantly lower 30-day mortality rate as patients who underwent conventional imaging and selective CT scanning after correcting for in-hospital GCS score.^6^ Given the traumatic head injury in our patient and resulting low GCS score, he was not able to localize symptoms or provide a history related to the trauma. A multicenter randomized clinical trial is currently underway to determine the value of immediate total-body CT scanning in trauma patients (REACT-2).^7^

## Discussion

The patient was diagnosed with a simple PAVM related to possible or suspected hereditary hemorrhagic telangiectasia (HHT). Simple PAVMs receive blood through a single artery with the afferent supply usually a branch of the pulmonary artery and the efferent limb originating within branches from the pulmonary vein.^1^ Based on the Curaçao criteria, a diagnosis of definite HHT is determined if at least 3 of the following are present: (1) epistaxis, (2) multiple telangiectasias at characteristic sites, (3) visceral vascular lesions, and (4) family history of a first-degree relative with HHT according to these criteria (Table 1). The disease is possible or suspected if 2 criteria are present, such as in our patient.^3^ The patient did not have any visible telangiectasias on physical examination or a family history of HHT, but on further interview he did endorse recurrent epistaxis. This coupled with the finding of a PAVM led to the presumptive diagnosis of HHT.

**Table 1.** Curaçao Criteria for Clinical Diagnosis of Hereditary Hemorrhagic Telangiectasia (HHT).^5^

Criteria	Description
Epistaxis	Spontaneous and recurrent
Telangiectasias	Multiple, at characteristic sites: lips, oral cavity, fingers, nose
Visceral lesions	Gastrointestinal telangiectasia, pulmonary, hepatic, cerebral, or spinal arteriovenous malformations
Family history	A first-degree relative with HHT according to these criteria

Patients with HHT usually present with recurrent epistaxis, iron deficiency anemia, and/or complications attributable to previously silent arteriovenous malformations. Age-related penetrance occurs in HHT so that increasing manifestations of the disease develop with aging.^4^ Thus, the International HHT Guidelines Working Group recommends that clinicians consider the diagnosis of HHT in patients with one or more Curaçao criteria. At least 90% of patients meet the Curaçao clinical criteria by age 40. The average age of onset for epistaxis is 12 years, with the appearance of telangiectasias of the mouth, face, or hands 5 to 30 years later. Approximately 23% of patients with HHT have a cerebral vascular malformation with a bleeding risk of 0.5% per year. Genetic testing can be performed to identify the causative mutation in a family with clinically confirmed HHT, in relatives of a person with a known causative mutation, or in individuals who do not meet clinical diagnostic criteria but need an established diagnosis. Testing involves DNA sequencing and deletion/duplication analysis of the coding exons of the endoglin gene (*ENG*) and the activing A receptor type II-like 1 gene (*ACVRL1*).^5^

PAVMs are present in 15% to 50% of people with HHT and have been associated with life-threatening complications, such as stroke, transient ischemic attack, cerebral abscess, massive hemoptysis, and spontaneous hemothorax.^8^ A right-to-left shunt that causes hypoxemia and paradoxical emboli is also one of the main complications. Transthoracic contrast echocardiography with agitated saline detects intrapulmonary shunting, showing a delayed appearance of microbubbles in the left side of the heart after 3 to 4 cardiac cycles.^1^ The test is recommended as the initial PAVM screening test due to its high sensitivity. If negative, then the family member would not need a thoracic CT scan, thus limiting exposure to radiation at the point of screening. Those with findings suggestive of shunting are followed with a chest CT.^5^ Transthoracic contrast echocardiography with agitated saline is also helpful in determining if a PAVM is amenable to embolization therapy.^8^

Untreated PAVMs pose a significant morbidity, including stroke, cerebral abscess, migraine, pulmonary hypertension, and hemorrhage.^1,2^ Embolization is considered first-line treatment of asymptomatic PAVMs to prevent these later complications.^9^ During embolization, the supplying artery immediately preceding the PAVM is the target to occlude with the ideal site just proximal to the aneurysmal sac; thus, the occlusion is achieved as distal as possible to avoid occluding branches to normal adjacent lung.^1^ Risks of the procedure include transient pleurisy; paradoxical embolism of devices, thrombi, or air bubbles (rare in incidence); and potential massive hemoptysis from development of systemic arterial collaterals that develop months after embolization.^2^ Feeding arteries with luminal diameters <2 to 3 mm can be technically difficult to treat; however, the introduction of microcatheter systems and detachable microcoils have made it possible to safely embolize some small PAVMs, even below 2 mm in diameter.^1,2,10^ If a PAVM is not able to be embolized, it can grow over time, leading to symptoms. At this point, a repeat attempt at embolization can be performed.^10^ Follow-up imaging can also be done within a 3- to 5-year period.^5^

Our patient underwent a pulmonary arteriography of the right upper and lower lobe with the use of a microcatheter system; however, the PAVM could not be visualized. The patient was educated on his diagnosis of a PAVM and the need to monitor for symptoms of growth and repeat chest imaging in 3 to 5 years. He was also counseled on the need for prophylactic antibiotics for future dental procedures because there have been links between oral bacteria, dental procedures, and PAVM-associated cerebral abscess.^11^ The patient and his family were informed of the possible or suspected HHT diagnosis. If the patient were to develop telangiectasias, then he would meet criteria for definite HHT and first-degree relatives would need screening. The patient improved neurologically with residual left-sided weakness and was started on warfarin for his venous sinus thromboses. After a 3-week hospitalization, he was transferred to a rehabilitation facility.

## Conclusion

Our young patient with a history of epistaxis was found to have a PAVM, which was incidentally identified on trauma imaging after a fall. He had a large subdural hematoma from the trauma but did not have any intracerebral hemorrhage or cerebral arteriovenous malformations that would be related to HHT. HHT should be considered if even one of the Curaçao criteria is present. Our case highlights the importance of obtaining a complete past medical and family history in young patients who have recurrent epistaxis as additional history may prompt suspicion for HHT. The diagnosis can be made clinically and directly affects family members, who would otherwise not receive appropriate screening.
